# A Comparative Study of Intralesional Acyclovir vs Immunotherapy for Treatment of Viral Warts

**DOI:** 10.7759/cureus.38781

**Published:** 2023-05-09

**Authors:** E. Meghana Reddy, T.S. Rajashekar, K. Suresh Kumar

**Affiliations:** 1 Dermatology, Sri Devaraj Urs Academy of Higher Education and Research, Tamaka, IND; 2 Dermatology, Venereology and Leprosy, Sri Devaraj Urs Academy of Higher Education and Research, Tamaka, IND; 3 Dermatology, Sri Devaraj Urs Academy for Higher Education and Research, Tamaka, IND

**Keywords:** human papillomavirus, ppd, intralesional purified protein derivative, intralesional acyclovir, viral warts

## Abstract

Background: Viral warts are caused by human papillomavirus (HPV), are difficult to treat with conventional modalities, and are cosmetically disfiguring; hence, immunomodulators are being used. The viral origin of warts suggests the antiviral drug acyclovir as a potential therapeutic option. The current study compares the effect of intralesional acyclovir (nucleoside analogue) and intralesional purified protein derivative (PPD) (immunotherapy) in treating various viral warts.

Methodology: Prospective observational comparative study was conducted to determine the efficacy of acyclovir, and PPD administered via the intralesional route in patients with viral warts. The study population was categorized into two groups. One group received intralesional acyclovir, and the other received intralesional PPD. Patients were followed-up with for three months. Outcomes considered in our study were recovery (complete, partial, and no recovery) and side effects like pain, burning sensation, and desquamation. Statistical analysis was carried out by coguide software.

Results: In our study total of 40 participants, 20 in each group were included. 25 and 15 were of age <30, and ≥ 30, respectively, while 20 were males, and 20 females. Our study reported 60%, and 30% of complete recovery with intralesional acyclovir treatment and intralesional PPD treatment, respectively, in the twelfth week. However, p-value > 0.05 represented no significance between groups. 90% in the acyclovir-treated group presented with pain, and 100% presented with burning sensation, while in the case of PPD-treated group, 60% presented no side effects and the rest 40% showed pain.

Conclusions: Intralesional acyclovir is more effective in treating viral warts than PPD. The focus is to be laid on anticipated side effects.

## Introduction

Viral warts are benign lesions caused by human papillomavirus (HPV) that cause pain, bleeding, and cosmetic disfigurement [1]. Acquisition of HPV is based on various factors, including the nature of the contact, the quantity of viral load, the HPV-specific immunologic status of the exposed individual the degree, and the location of lesions [2,3]. Its recurrence and persistence affect quality of life. The prevalence of cutaneous warts is 7-12% approximately [4,5]. About two-thirds of warts resolve on their own, but most patients seek treatment because of their rapidly increasing number, associated tenderness, and unsightly appearance [4,5].

Warts can be treated with the help of various therapeutic modalities. Primary therapies include cryotherapy, topical salicylic acid, excision, laser vaporization, electrocautery, topical imiquimod, and topical 5-fluorouracil. In recent times, the promising modality seems to be immunotherapy, as it has been found that, in wart resolution, a major role is played by cell-mediated immunity (CMI), which underline the need for immune protection against infection caused by HPV [6-8]. Some of the immunotherapy agents that have been tried include imiquimod, cimetidine, interferons, candida albicans antigens, tuberculin purified protein derivatives (PPDs), and measles, mumps, and rubella (MMR) vaccines [9,10]. Among the present immunotherapy agents, intralesional PPD is more effective and well tolerated mode of immunotherapy as it prevents recurrence of warts [11,12]. Despite having a variety of therapeutic approaches, none of these treatments work as antivirals and do not selectively destroy cells that are infected by the virus. Therefore, there is a constant need for the creation of an antiviral therapy that can target, and kill the virus, particularly in the HPV-infected tissue [13].

Acyclovir is a synthetic analogue of a purine nucleoside that has antiviral action against some viruses, including varicella-zoster and herpes simplex. Acyclovir is converted to its active form, acyclovir triphosphate, by virus thymidine kinase, which specifically targets viral DNA to inhibit viral replication in the host. Very few studies and case reports have previously described the treatment of warts using both topical and oral acyclovir, some of which were resistant to prior treatment [14-17]. In recent research, the effectiveness of intralesional acyclovir versus intralesional saline in treating warts was compared. The results showed that acyclovir had a statistically significant complete clearance rate of 52.6% complete clearance, suggesting it to be a potential treatment option [18]. Intralesional acyclovir is a very recently used modality in treatment, and only very few studies have been done to assess the efficacy of acyclovir. So, we have undertaken this present study to assess the efficacy of intralesional acyclovir compared with widely accepted treatment modality immunotherapy using PPD in terms of recovery from viral warts.

## Materials and methods

Patient Selection

In this retrospective observational study, 40 patients who were clinically diagnosed with warts were screened and assessed in detail by the outpatient departments of Dermatology, Venereology, and Leprosy in a hospital in Karnataka. Patients with less than 10 warts with palmoplantar common, and/or peri-ungual presentations were included in the study. Patients under any topical or systemic treatment of warts for the last four weeks and pregnant or lactating women were excluded. Patients who were immunocompromised, with active TB and/or history of TB, or with keloidal tendency were also excluded. Children less than 12 years of age and those who refused to give consent were excluded from the study. The institutional ethical committee approved the study (Reference No: DMC/KLR/IEC/93/2022-23), and before the administration of injections, written informed consent was taken from all patients with their signatures. All patients fulfilling the inclusion criteria were categorized into two groups depending on the treatment they received, namely, Group A (intralesional PPD) and Group B (intralesional acyclovir) (n=20 per group)

Treatment Protocol

In Group A, the single largest wart in each case was selected, and 0.1 ml of PPD antigen (Tuberculin PPD (ARKRAY)), was injected into it using an insulin syringe, which was repeated every two weeks to a maximum of six sessions (0, 2, 4, 6, 8, 12 weeks) on the same or different wart, based on the reversion of the wart. Intralesional injection of acyclovir (70 mg/ml) (Acivir (Cipla)) was used to treat Group B, with a dose of 0.1 ml injected into each wart at the base. The injection was repeated every two weeks to a maximum of six sessions (0, 2, 4, 6, 8, 12 weeks). If, at follow-up visits, complete resolution was seen, treatment was discontinued.

Assessment of Response

The number and distribution of warts, and effects of local side effects were noted at 0, 2, 4, 6, and 8 weeks. A continuous clinical photographic record was maintained. The response was evaluated as follows: Complete response: clearance of all warts completely; Partial response: reduced number or size of warts (but not completely disappeared); and No response: no reduction in the number and size of lesions. Warts are classified into different types as shown in Table 1. Adverse effects (constitutional or local symptoms, if present) were noted. Patients who showed complete response were followed up for three months to note any recurrence or residual skin changes. The collected data were analyzed as per the statistical plan.

**Table 1:** Types of warts

Types of warts	Description of warts
Common wart	A small fleshy bump on the skin most often on fingers or hands
Palmar wart	Noncancerous skin growth caused by a viral infection on the top layer of the skin
Plantar wart	Hard, grainy growth on the heels or balls of the feet
Periungual wart	Found around the nails, common in nail biters
Subungual wart	Occur underneath the fingernail

Statistical analysis

Therapeutic response at different time points was compared between two treatment modalities using the Chi-square test. The type of wart was reported in two treatment modalities across the therapeutic response. P value <0.05 was considered statistically significant. coGuide Statistics software, Version 1.0 was used for Data analysis [19].

## Results

A total of 40 subjects were considered in the study. Out of that, 20 received intralesional injection acyclovir and 20 received intralesional injection PPD. In the <30 age group, 12 (60%) participants received intralesional injection acyclovir and 13 (65%) received intralesional injection PPD, and in ≥30 years age group, eight (40%) participants received intralesional injection acyclovir and seven (35%) received intralesional injection PPD. In the group receiving intralesional injection acyclovir, 12 (60%) were male participants and eight (40%) were female, and in the group receiving intralesional injection PPD, 11 (55%) were male and nine (45%) were female participants. In Group B, the majority of six (30%) participants had a common wart, four (20%) had a palmar wart, and six (30%) had a plantar wart. In Group A, the majority of seven (35%) had a common wart, five (25%) had a palmar wart, and four (20%) had a plantar wart. 

In our study, 25 participants were of age <30 years, of which 12 and 13 were in Groups B and A, respectively, and 15 participants were of age ≥30 years, of which eight and seven were in Groups B and A, respectively. There were 20 males in the study, of which 12 were in Group B and eight were in Group A. Of 20 females, 11 were in Group B and nine in Group A (Table 2).

**Table 2:** Demographic details of study population (n=40)

Parameter	Intralesional Injection Acyclovir	Intralesional Injection PPD
Age		
<30	12 (60.0%)	13 (65.0%)
≥30	8 (40.0%)	7 (35.0%)
Gender		
Males	12 (60.0%)	8 (40.0%)
Females	11 (55.0%)	9 (45.0%)
Type of warts		
Common warts	6 (30.0%)	7 (35.0%)
Plantar warts	6 (30.0%)	4 (20.0%)
Palmar Warts	4 (20.0%)	5 (25.0%)

In Groups B and A, six (30%) and three (15%) participants reported partial response at two weeks, respectively. Complete response was seen in 12 (60%) and six (30%) participants in Groups B and A at 12 weeks, respectively. The difference in the proportion of therapeutic response between the two treatment modalities was statistically not significant in the 2nd, 6th, 10th, and 12th week with P value >0.05, where it was shown a significant difference in the 4th week (P value <0.05) (Table 3).

**Table 3:** Comparison of therapeutic response rate in intralesional injection acyclovir and intralesional injection PPD groups (n=40) PPD:

Parameter	Treatment		Chi- square value	P value
	Intralesional Injection Acyclovir (n=20)	Intralesional Injection PPD (n=20)		
2 weeks				
Partial Response	6 (30.0%)	3 (15.0%)	1.29	0.4506
No Response	14 (70.0%)	17 (85.0%)		
4 weeks				
Partial Response	13 (65.0%)	5 (25.0%)	6.46	0.0110
No Response	7 (35.0%)	15 (75.0%)		
6 weeks				
Complete Response	3 (15.0%)	0 (0.0%)	*	*
Partial Response	12 (60.0%)	6 (30.0%)		
No Response	5 (25.0%)	14 (70.0%)		
8 weeks				
Complete Response	7 (35.0%)	2 (10.0%)	5.41	0.0669
Partial Response	9 (45.0%)	8 (40.0%)		
No Response	4 (20.0%)	10 (50.0%)		
10 weeks				
Complete Response	10 (50.0%)	4 (20.0%)	4.91	0.0858
Partial Response	7 (35.0%)	8 (40.0%)		
No Response	3 (15.0%)	8 (40.0%)		
12 weeks				
Complete Response	12 (60.0%)	6 (30.0%)	3.93	0.1399
Partial Response	5 (25.0%)	7 (35.0%)		
No Response	3 (15.0%)	7 (35.0%)		

**Figure 1:**
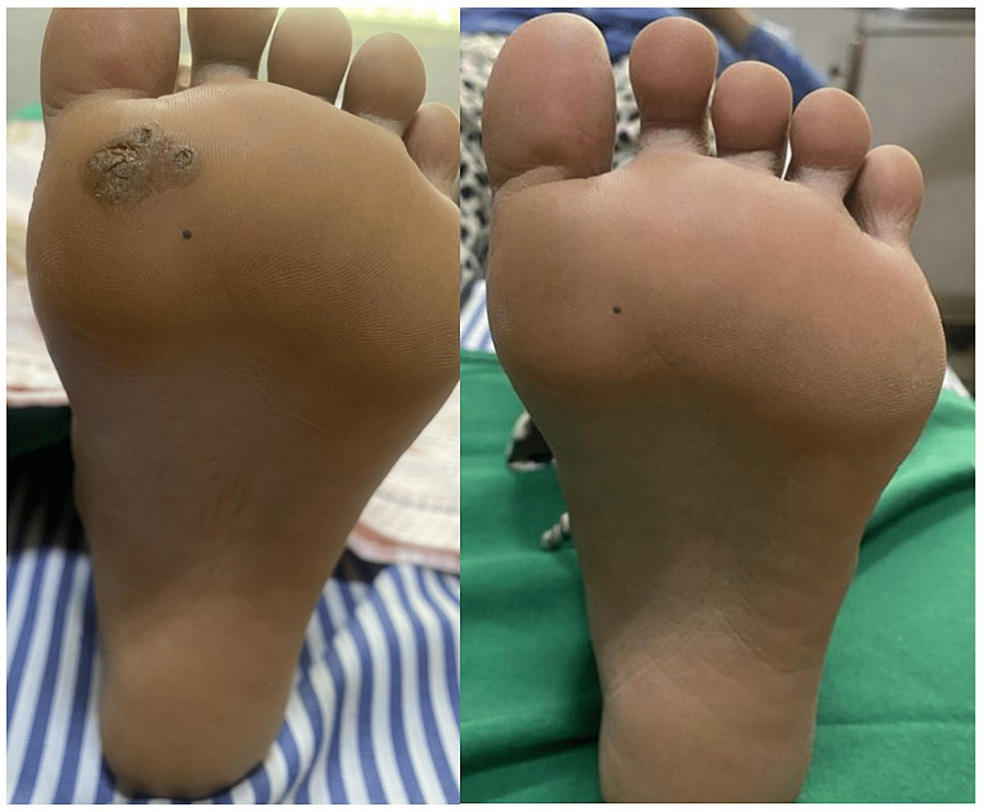
A 20-year-old female with plantar warts for one month showed complete resolution after treatment with four doses of intralesional injection acyclovir

**Figure 2:**
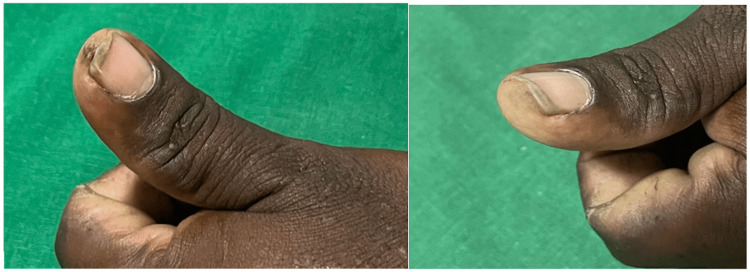
A 35-year-old male, with a subungual wart for six months was given three sittings of intralesional injection acyclovir, and showed complete resolution.

**Figure 3:**
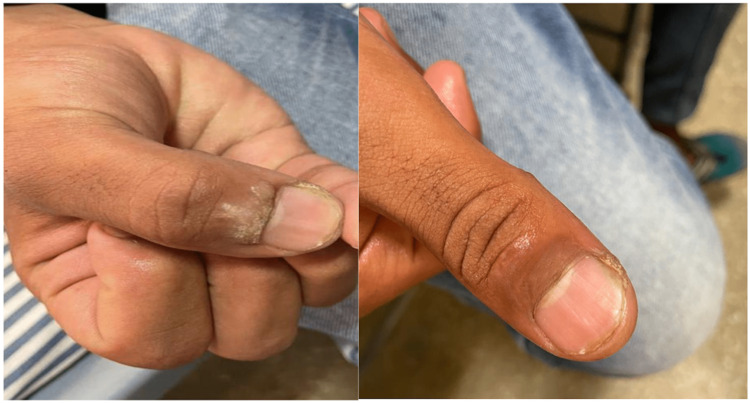
A 27-year-old male, with periungual wart for two months was given four doses of intralesional injection acyclovir, and showed complete resolution.

In Group B's cases of common warts, complete response was present in three (25%) in the 12th week; in the case of Palmar warts and plantar warts, complete response was in four (33.3%) participants each, whereas in periungual warts and subungual warts, only one (8.33%) participant each showed complete response (Table 4). Figures 1, 2, 3 compare plantar, subungual, and periungual warts before and after treatment with intralesional injection acyclovir respectively.

**Table 4:** Comparison of type of warts with 12 weeks for Group B (n=20) *No test is applicable due to the 0 cells of the data.

Type of Wart	At end of 12 weeks			Chi- square value	P value
	Complete Response	Partial Response (n=5)	No Response (n=3)		
Common wart (n=8)	3 (37.5%)	2 (25.0 %)	3 (37.5%)	*	*
Palmar wart (n=7)	4 (57.1%)	2 (28.5%)	1 (14.2%)	0.07	0.964
Plantar wart (n=6)	4 (33.3%)	2 (40.0%)	0 (0%)	*	*
Periungual wart (n=1)	1 (100%)	0 (0%)	0 (0%)	*	*
Subungual wart (n=1)	1 (100%)	0 (0%)	0 (0%)	*	*

**Figure 4:**
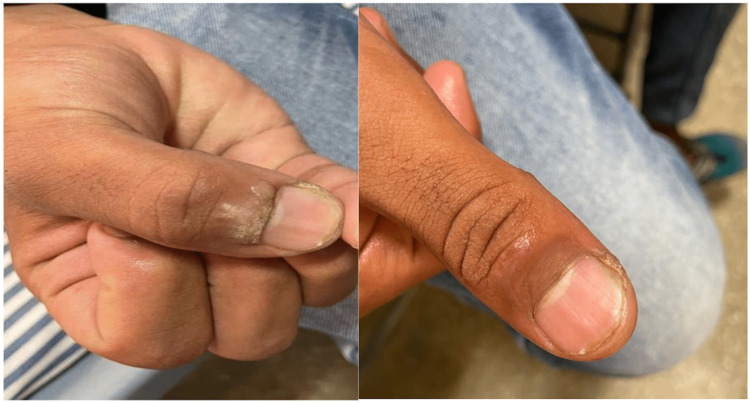
A 27-year-old male, with periungual wart for two months was given four doses of intralesional injection acyclovir, and showed complete resolution.

In Group A, in the case of common warts, complete response was seen in three (37.5%) by 12th week. In the case of Palmar warts and plantar warts, complete response was in four (28.5% each). In periungual warts and subungual warts, one (100%) participant responded completely (Table 5). Figure 4 compares plantar, subungual, and periungual warts before, and after treatment with intralesional injection acyclovir respectively.

**Table 5:** Comparison of type of warts with 12 weeks for Group B (n=20) PPD: Purified protein derivative * No test is applicable due to the 0 cells of the data.

Type of wart	12 weeks			Chi- square value	P value
	Complete Response	Partial Response	No Response		
Common wart (n=8)	3 (37.5%)	3 (37.5%)	2 (25.0%)	0.65	0.7208
Palmar wart (n=7)	2 (28.5%)	3 (42.86%)	2 (28.57%)	0.32	0.8503
Plantar wart (n=7)	2 (28.5%)	1 (14.29%)	4 (57.14%)	2.84	0.2422
Periungual wart (n=0)	0 (0.0%)	0 (0.0%)	0 (0.00%)	*	*
Subungual wart (n=1)	0 (0.0%)	0 (0.0%)	1 (100%)	*	*

 

In the PPD treatment, 12 (60%) participants had no side effects, and eight (40%) participants reported pain. In the acylcovir treatment, 20 (100%) participants reported a burning sensation, three (15%) reported desquamation, and 18 (90%) reported pain (Table 6).

**Table 6:** Side effects noted in PPD (n=20) and acyclovir (n=20) groups PPD:

Treatment	No side effects	Burning sensation	Desquamation	Pain
PPD (n=20)	12 (60.0%)	0 (0%)	0 (0%)	8 (40.0%)
Acylcovir (n=20)	0 (0%)	20 (100%)	3 (15.0%)	18 (90.0%)

## Discussion

The present prospective comparative study conducted on the treatment of viral warts reported 60% of complete recovery in the case of treatment with intralesional acyclovir, only 30% in case of treatment with intralesional PPD, and 25%, and 35% of the partially recovered population respectively. No response was found only in 15% of patients treated with injection acyclovir, and 35% of patients were treated with injection PPD in the 12th week. However, p-value > 0.05 represented no statistically significant difference between groups. The greater response seen in patients treated with intralesional acyclovir can be attributed to its specific antiviral action explained by possible cellular enzymes phosphorylation of the drug that inhibits viral DNA integration. In addition, HPV-infected cells apoptosis may be induced.

In another study, the efficacy of intralesional acyclovir is compared with intralesional saline (control), which reported 52.6% complete recovery, 36.8% partial recovery, and 10.5% no recovery upon patients treated with acyclovir, which falls in line with our current study findings [20]. But in the same study, highly significant statistical difference (p-value < 0.01) in the outcome seen between groups contradicts the current research findings where the results do not have any significant difference. This contraindication in the results between two studies is due to the methodology adopted in the both studies: In the cited study, the acyclovir-treated group is compared with the saline-treated group, whereas in the present study, it is compared with the PPD-treated group [18]. In contrast, another study comparing liquid nitrogen with acyclovir cream with placebo cream for the treatment of plantar warts reported no beneficial effects of acyclovir cream over placebo cream for the treatment of plantar warts [21]. This is due to the difference in the mode of drug delivery as intralesional mode delivery has a higher concentration of the drug delivered directly into the lesions without any significant systemic absorption, and also has deeper penetration bypassing the superficial barrier zone. Another comparative study comparing the efficacy of intralesional PPD with BCG strain in the treatment of cutaneous warts reported similar findings of intralesional PPD treatment in our study [22].

In patients treated with injection acyclovir diverse response was seen in different types of warts, the maximum response was seen in periungual warts and subungual warts, which showed absolute resolution in 100% of cases. This is followed by complete clearance of palmar warts seen in 57.13% of the cases. Complete resolution is seen by common warts and plantar warts in 37.5%, and 33.3% of the cases, respectively. When compared to injection PPD, where no response is seen in subungual warts and complete response in common warts, palmar warts, and plantar warts, we can infer that intralesional acyclovir is a better option for treating periungual and subungual warts, which showed positive results.

When it comes to side effects profile, intralesional acyclovir in our current study reported burning sensation in 100% population and pain in 90% population as side effects, which is similar to another study that reported the side effects of intralesional acyclovir in 93.8% of the patients as experience of pain during injection. Contrary to other studies for the treatment of genital herpes infection, topical acyclovir was used, and reported no side effects or adverse effects as seen in studies [22,23]. Our current comparative study on the treatment of viral warts using intralesional acyclovir and PPD reported beneficial effects of acyclovir with complete response seen in the majority of patients, and no response in the minor group of the population compared to patients treated with PPD at end of 12 weeks. This study is a non-randomized trial with less sample size, which we consider as a limitation of the study. We would recommend that future studies conduct randomized control trials to postulate the benefits of acyclovir over PPD with a greater sample size.

## Conclusions

In treating viral warts, a therapeutic response full of promise was demonstrated by intralesional acyclovir, particularly for periungual and subungual warts, without causing any severe or life-threatening side effects. The focus should be placed on the side effects when deciding on patients who appear with viral warts.
